# Tetanus

**Published:** 1886-04-20

**Authors:** H. F. Scott

**Affiliations:** Atlanta, Ga.


### Southern Medical Record.

A MONTHLY JOURNAL OF PRACTICAL MEDICINE.

☞All Communications must be addressed to R. C. WORD, M. D., Managing Editor.

"*Quicquid Præcipies Esto Brevis.*"

VOL. XVI. ATLANTA, GA., APRIL 20, 1886. NO. 4.

### ORIGINAL AND SELECTED ARTICLES.

### TETANUS.

BY H. F. SCOTT, M. D., OF ATLANTA, GA.

[Read before the Atlanta Society of Medicine, March, 1886.]

It so happened that soon after receiving my first medical diploma, I was called to assist a brother physician in the management of a case of traumatic tetanus, and I was very much impressed with its gravity and the paucity and inefficiency of medical resources in such cases.

The case was that of a wood-chopper, who, some two weeks previously, had run a nail in the sole of his right foot, and had, as he thought, withdrawn all of it. Some days thereafter he was seized with tetanus of a most violent nature, for which morphine internally and subcutaneously was tried without any great relief. Hydrate of chloral, which had just come into use, also proved unsatisfactory.

The attending physician, suspecting the existence of a piece of nail imbedded in the foot, asked me to assist him in the search for it. There being such tenderness of parts the least handling sufficed to bring on spasms of the most violent nature. We therefore proceeded to chloroform the patient. He had only taken a few whiffs when a violent paroxysm occurred, followed by spasm of glottis and upturning of eyes, and the patient ceased to breathe, though a feeble heart-beat continued for a few minutes. Every method of resuscitation known to us, except the use of a battery, which was not to be had at that moment, was tried without avail. Afterwards, we made an incision and found the piece of nail imbedded in the tissues of the foot, between the metatarsal bones.

Before proceeding to discuss the appropriate treatment of tetanus, let us consider what is known as to its etiology, symptomatology, etc.

In tetanus there is an uncontrollable tonic spasm of the muscles of the body ; either the entire muscular system or the greater portion thereof being affected. In those cases where the spasms are confined to the muscles of the jaw, we denominate it trismus, or lock-jaw. As a rule, trismus is only one of the many symptoms of tetanus, though under certain circumstances—for example, in teething children, or in diseases of the jawbone and its connections—it may be wholly a local process.

The most practical division of tetanus would, probably, be as follows : *T. Traumaticus*, where it follows an injury, and, *T. Rheumaticus* or *Idiopathicus*, where no discoverable cause for the production of the affection exists, or where simply a catching of cold is assumed.

Aside from the original cause of their setting up, there exists absolutely no fundamental difference between these two classes ; the symptoms in both are identical. Both classes may pursue an acute or chronic course, though traumatic cases are generally acute and may be rapidly fatal, while the idiopathic are oftener chronic, and their prognosis is much more favorable. As predisposing to the development of this affection must be considered, in general, everything which greatly reduces the bodily energy or strength ; for example, typhus and the abuse of alcoholic liquors are considered by many authorities as prolific causes.

Tetanus is more common in man than in woman, and this, undoubtedly, is due to the greater hardships and exposure incident to men's occupations than is the case with women's pursuits. It is a well-known fact, that more than half of all cases occur between ten and forty years of age, just in the most active and exposed period of a man's life.

This affection is met with about equally summer and winter, but is much oftener found in warm than in cold climates ; the coasts of the tropics, where sudden changes in temperature occur—the exceedingly hot days being followed by cool nights—are especially often visited by this disease. It is affirmed to be more frequent and more grave in negroes, but this has not been my experience.

The wounded, after long and severe battles, are especially prone to tetanus ; thus we have the testimony of the celebrated Larrey that one hundred cases developed on the morning succeeding a battle.

As to direct traumatic causes, as is well known, it may follow any injury, from the most insignificant scratch to the severest complicated fracture, after the extraction of a tooth as well as following the severest surgical operation. Still we most often find it following severe wounds and injuries—for instance, burns, frost-bite, complicated fractures, and especially after wounds in tissues, such as the hands and feet, that are very delicate and abundantly supplied with nerves.

Traumatic tetanus usually ensues within the first or second week after exposure to the injury. Should no symptoms occur before the third week, there is slight probability of its appearance.

After all the injuries mentioned above, we should be on the outlook for its development; but it must be admitted there are no *positive* general or local symptoms by which its commencement can be recognized. Usually, the patient first notices that during talking or eating he experiences a difficulty in opening his mouth on account of stiffness of the muscles of the lower jaw—the attempt to do so being, often, accompanied by sharp and drawing pains; still this symptom may be so slight as scarcely to be complained of. The first sure indication of the presence of tetanus is a hardness and spasmodic contraction of the muscles of the neck, back or abdomen; at the same time the countenance begins to assume a peculiar drawn and fixed appearance, due to contraction of some of the muscles of the face. Should a drawing down of the corners of the mouth ensue, we have the so-called "tetanic grin," or *risus sardonicus*. Then ensues a difficulty about swallowing, so that at each attempt to drink there is a spasm of the muscles of deglutition, and even of respiration. Gradually the entire muscular system may become affected, the tetanic cramps manifesting themselves, sometimes more in muscles of the trunk, at others in those of extremities; though the muscles of back of neck and trunk are most usually affected. Following Larrey's leadership, these latter have been divided into different forms, according as certain muscular groups are affected. Thus we have Opisthotonos, Pleurosthonos, and Emprosthotonos, where the body is drawn backwards, to one side, or forwards—opisthotonos being the most frequent variety.

Tetanus may be confounded with certain other affections, whose symptoms are very similar, and in some instances their differential diagnosis is very difficult; for instance, tetanus and strychnia poisoning. I will only mention two points of difference, as the remainder will readily suggest themselves. The former is a progressive disease, while the latter appears suddenly in all is severity. In the first there is a constant stiffness of the muscles of the jaw ; while in the second the jaw is only locked during the spasm. Any one, never having seen a case of hydrophobia, would likely confound it with tetanus ; yet the peculiar restlessness of mind and body in the former, the entire intermission of spasm at intervals, and the well-known aversion to fluids, with the most excessive thirst present, would be sufficient to distinguish between them. *En passant* it may be noted that cases are reported where these two troubles were present in one and the same case at the same time. The fact that the patient's mental faculties remain unimpaired—any delirium being seldom observed—would aid in distingulshing it from cerebro-spinal meningitis.

Authorities vary in their statements as to the temperature in tetanus. Neudorfer affirms it to be the only instance of an acute affection where there is no rise in temperature, while Billroth and others state there is much rise, especially in most acute cases, where it often reaches 107°. As a rule, chronic cases show no rise in temperature. It is a remarkable fact that, after death, it rises, sometimes to a high degree (113°), but the reason for this is undecided. The patient's mental faculties are unimpaired, any delirium being seldom observed.

In treating tetanus, we should bear in mind the fact that acute cases die from apnœa, through spasm of the muscles of respiration, more particularly the laryngeal muscles.

Now, it is generally admitted that, once well developed, the disease will run its course, and, farther, that our efforts to a great extent should be directed to preventing suffocation. For this purpose, tracheotomy is highly recommended, and, if undertaken sufficiently early, seems worthy of our adoption, for the reason that, with a tube in the trachea, death by laryngeal spasm cannot occur, and hence there is a better prospect of recovery under other appropriate treatment. *Per contra*, chronic cases prove fatal from exhaustion. Here our efforts must be directed to sustaining the patient's strength, by judicious feeding by the mouth, if practicable; if not, by nutritious enameta, repeated every four to six hours.

For the purpose of securing to the patient the needful mental and physical quietude, perhaps every known narcotic and anæsthetic has been tried, and praised. Opium, especially morphia hypodermically, is highly recommended. Demarquey recommends its injection deep into the drawn muscles ; for instance, elevator muscles of the jaw, and sterno-cleido-mastoideus, muscles of the back of neck and body. Chloroform inhalations are much used ; the patients are greatly relieved by being thoroughly brought under its influence; but we should always remember the natural tendency to spasm of the glottis, and be guarded in its adminstration. Hydrate of chloral is perhaps best woithy of our confidence, as many cases are reported as recovering after its use in large doses; and it may be readily given per enema where circumstances demand it. Authorities differ as to the value of cirraria and eserine. Ice-bags along the spine are highly recommended by Hammond and others. I believe hyoscyamin would be a useful agent—given in 1-16 to 1-12 grain hypodermically, when required.

Before concluding, I desire to direct attention to the appearance and condition of the nerves leading to and from the point of injury in traumatic cases, and also to the condition of the spinal chord; for the reason that I am a firm believer in the "nervous theory" of this affection—according to which some known or unknown peripheral irritation exerts its influence upon the nerve filaments, and is conducted along the nerve trunks to the spinal chord—thereby inducing a state of exaggerated functional activity in the latter, as evidenced by muscular contractions, etc.

In most cases, where patients have sufficiently long survived, we find neuritis and perineuritis of these nerves, they being soft and swollen, and the neurilemsua being hyperæmic, and often extravasation of blood within nerve-sheaths. These changes gradually proceed up the nerve, becoming more and more evident the longer the initial irritation lasts, until they reach the spinal chord, which may show similar changes in the substance and coverings, most marked where the nerves leading from affected parts enter the chord.

Now, granting the above statements to be true, I consider the most rational and worthy course in all traumatic cases would be, first, to carefully cleanse the wound, and search for and remove any foreign body, and treat in a strictly antiseptic manner; at the same time dividing any torn or bruised nerves. Further, as soon as the least symptom of impending tetanus is manifest, a portion, or all, of the nerves of the affected limb should either be elongated, preferably combined with crushing, or neurotomy should be done. These procedures are recommended for the purpose of at once and thoroughly cutting off all communication between the injured part and the central nervous system, and thus avoiding the continual irritation and excitation of that system. It is self-evident that these procedures, to be of much service, must be early resorted to—before the long-continued peripheral irritation has brought about such deep-seated alterations in the central nervous system as to keep up the trouble even after the removal of the initial cause, and prevent its yielding to general treatment. But, to be secure, these operations on the nerves should be undertaken at a considerable distance from the wound, instead of in its immediate vicinity, as is usually recommended, and done, for in no other way can we be sure of getting beyond the affected portion of the nerves. As the end aimed at is only the temporary interruption of a nervous conductibility between the chord and peripheral irritation, simple stretching of the nerves, with or without crushing, would probably fulfill that indication in most cases, and would leave no bad after effects.

In 1877, I insisted, in my thesis presented to the faculty of the Frederick-William University for a diploma in that Institution, that neurotomy, or nerve-section, at a remote distance from the point of injury offered the safest and most rational and surest mode of dealing with these cases. Where special signs pointed to the implication of only one nerve, it alone should be divided ; otherwise total neurotomy, or true nervous amputation of the limb, as it has been called, should be resorted to, and thus perfect rest secured. Total neurotomy would certainly leave a nearly useless and paralyzed limb ; but in the course of six months this paralysis might, to a great extent, disappear; especially if, in dividing the nerves, we are careful to leave the severed ends in apposition, and not handle them roughly, might we expect this result.

Immediate union of divided nerves *never* occurs, but they are soon united by connective tissue, if not too widely separated (not over 2¾ inches), and ultimately by a process of "sprouting" nervous conductibility is re-established. Where only a few of the nerves of a limb are divided, nervous connection is said to be soon re-established through anastomising branches

The operation of nerve-section is very simple, and union by first intention should readily follow. Granting that permanent paralysis may ensue, still neurotomy is infinitely preferable to amputation of the affected part, as recommended by Larrey, Petit, and others—Petit declaring it to be a sure cure, if done at earliest manifestation of the affection. Should nervous communication fail to be re-established, we possibly might find a solution of the difficulty by paring the ends of the disunited nerves and bringing them together by nerve-suture.

